# Dr. Kinglake in Reply to Mr. Harrold, on Burns and Scalds

**Published:** 1806-07

**Authors:** Robert Kinglake

**Affiliations:** Taunton


### Dr. Kinglake in Reply to Mr. Harrold, on Burns and Scalds.

*To the Editors of the Medical and Physical Journal.*

Gentlemen,

It is without reluctance that I enter into a controversial discussion with Mr. Harrold, on the subject of burns and scalds, as he appears to be actuated both by sentiments and principles in his philosophical inquiries, much too correct to endanger any illiberal consequence from the collision of our opinions.

The reason which Mr. H. states for being dissatisfied with cold applications in cases of burns and scalds is, that "he had too frequently seen the ill effect" of them. My reason for vindicating them is, that they have never *failed* in my observation, to an extent that could warrant me in resorting to any other remedy. It was not my design so much to object to the opposite mode of treatment, as to defend the topical use of cold water from the unqualified and unmerited reproach charged on it. Dr. Kentish's terebinthinate remedy, as well as Mr. H's explanation of its mode of operating, shall be adopted by me the moment that cold water shall cease to realise every benefit that could be wished. Until then, the partizans of the opposite treatment must not attempt, by hypothetical argument, to eclipse the luminous evidence of my senses; to persuade me that demonstrative proof is in reality a delusion, a mere phantom of imagination.

Were your Journal at liberty to report a large variety of instances of the direct and curative efficacy of topical cold in burns and scalds, it would not be difficult for me to furnish them in ample abundance; but surely, such examples would be deemed superfluous, too trite and common placed to find admission in a repository of original and novel intelligence. One fact by your permission I will relate, as it is of recent date, and occurred in my own family. A female servant occupied in the employment of washing, in transferring a pailful of boiling ley from one vessel to another, by accident fell, and received the contents of the pail on one leg, from the knee to the foot. The torture was extreme. The leg was instantly immerged into a pail of cold water, and there retained for a few minutes. The pain pain immediately became inconsiderable. The stocking after a short time was removed, when vesication was apparent throughout the whole extent of the scalded part. The accident occurred towards the evening; the patient soon after retired to bed, was provided with a deep vessel full of cold water, and was desired to keep cloths wetted in that fluid on the part during the night, renewing them as often as they might become warm. Finding immersion more effectual in allaying the pain, the patient resorted to it about the middle of the night, by sitting on the bed-side and putting the leg into the vessel of cold water. The relief now became so complete, that sound sleep *inadvertently* ensued; this unconscious state of tranquillity was rendered sufficiently commodious by *involuntarily* lying down on one side at the edge of the bed. In this situation the patient remained with the leg in the cold water until about six o'clock in the morning, which was her usual time of rising. The extremity was therefore uninterruptedly kept in cold water during a period of about six hours. In awaking, the patient was equally surprised and gratified at finding that an accidentally protracted application of the cooling remedy had effectually subdued all pain and inflammation. The injured part retained a slight redness, the vesicated cuticle was empty, unbroken, and wrinkled. The patient resumed her usual avocations the following day, and had no further complaint than that of a slight sore at the heel, caused by the pressure of the shoe. This scald was seen by several persons who were conversant with accidents of that nature, treated not indeed with the terebinthinate remedy, but in the usual manner, by olive oil and what are esteemed cooling ointments, and they were astonished at the rapid and perfect cure which had been effected by the *sole* application of cold water. This case stands not alone, it is the common domestic practice in this neighbourhood on the receipt of a burn or a scald, directly to plunge the injured part into cold water; and in as far as any thing is known to me to the contrary, the relief has been instantaneous, and the cure as speedy as could be reasonably desired. Thus much for practice.

It is however strange, *passing* strange, that what succeeds under the management of one person, should fail in the same physical and moral circumstances under that of another. Mr. H. stands too high in my estimation for veracity, and has too strong a claim to my confidence to admit of my doubting the sincerity of his professions. He believes himself to be correct, I believe myself to be so likewise.

likewise. Where then is the fallacy? Certainly not in the nature of things, but in the fallible reasoning of such fallible creatures as Mr. H. and myself. I shall hereafter review my practice in the use of topical cold to burns and scalds, and shall with scrupulous fidelity restate my opinion of the treatment. Mr. H. will make no undue concession to the doubt in question, by doing the same. This procedure may at length mutually convince us where the error lies, and that too much more decidedly than is likely to be effected by speculative discussions of the subject.

Mr. H. justly remarks, "If any man will be hardy enough to reason against facts, I do not see upon what ground he can be met. I must suppose that the principal object of a liberal writer is an inquiry after truth. Doctor Kinglake has, I think, been attacked unfairly; but why should he employ the same unfair weapon? The evidence of a respectable man ought not to be questioned until the experiment has failed in the hands of another; for, after all, experience must decide, and every mode of reasoning must prove futile without its support."

These sentiments are worthy of Mr. H. they should be made the *polar star* of scientific researches. But how do they apply to my conduct? My objections to the heating mode of treating the injury under consideration are offered in defence of a remedy which, in my experience, has been found adequate to every curative purpose; to rescue its just fame from the charge of its being accessary to the death of a patient in certain difficult cases referred to by Doctor Kentish, if it were adopted in bar of spirit of turpentine, which is contended to be *alone* infallible in saving life when imminently endangered by burns or scalds. If this is "truth," it is so only to Dr. Kentish, Mr. Harrold, and other advocates of the doctrine. It is not *truth* to me. The prompt, decisive, and adequate virtue of topical cold is the "truth that is the object of my inquiry," and which cannot be invalidated by any *theory* that may be opposed to it. *Facts, indisputable facts,* only can determine the question at issue.

Mr. Harrold refuses to "compliment" me for my analogy of a *runaway* horse," saying, "it appears to him to be infinitely more fanciful than just, and to have no other relation to the subject in question than matter has to mind." It were unreasonable in me to desire a stronger relation in support of my simile of a "runaway horse," than Mr. H. unconsciously concedes, in asserting, that it has no other connection than matter has to mind. To state the inseparable quality of the connection subsisting between matter and mind, would be to exemplify the strongest instance of reciprocity of power that could possibly be adduced. No theory of animated nature can dispense with this sort of connection. It is the ground of all efficient power, it constitutes the cause and effect on which depend the infinite phenomena of the physical world. Surely then the comparison of the "runaway horse" may be applied in illustration of the excitable and stimulating powers of organic life, much more appositely than Mr. H. would insinuate by his erroneous idea respecting the relation obtaining between matter and mind.

In opposition to my opinion, that gradually reducing inflammatory excitement by withholding a small portion only of the stimulating violence is likely to occasion gangrene and to terminate in death, Mr. H. charges me with having "evidently confounded the violence of the exciting cause with the violence of the consequent action." Where is this confusion Mr. H. alludes to? "The exciting cause" in the subject under discussion is *spirit of turpentine*, the "consequent action" is the inflammation it sustains. No *confusion* appears to be here made of cause and effect; but violent action is very naturally considered as resulting from a violent exciting cause, all which, in my apprehension, is of pernicious tendency.

Mr. H. now avails himself of the utmost latitude of analogy, in supposing that because the effects of excessive cold are best remedied by a *slow* application of heat, so likewise are those of immoderate heat or excitement by *gradually* diminishing the power of the exciting cause. The cases however do not appear to be parallel; The influence of excessive cold on the living animal fibre reduces the active powers of life to a state bordering on death; such a state of vital power is too feeble to admit of violent excitement without risking a mortal exhaustion of its sinking remains. A slow augmentation of exciting power in such circumstances is therefore most safe, and best adapted to restore and establish the energies of life. But does the converse of this reasoning hold with respect to a part suffering from a burn or any other similar cause, to a degree as to be verging on gangrene? Would it be expedient on such occasions to withhold or abstract no more of *stimulant violence* than it would be right to add of *heat* for the removal of numbness from excessive cold? Such procedure would protract the duration of inflammatory disease, counteract the natural tendency to a diminution tion of its violence from the loss of motive power, which its continuance necessarily occasions, and finally endanger the exhausted or lifeless condition of fibre which constitutes the gangrenous state.

To put the familiar instance of visceral inflammation occasioned by either the stimulus of distension or by any other hurtful power, ought the morbid excitement to be removed *gradually* to insure recovery? In thus graduating the agency of stimulating power, it should be remembered that a slight reduction only of inflammatory excitement would permit the disease to exist long enough eventually to destroy life, as certainly as might be effected by its acute or undiminished violence. Would it not therefore be most advisable at once to enter on the great work of avoiding the concomitant danger, by removing with the utmost rapidity the morbid excess of excitement. Fevers may be reduced; inflammations may be mitigated; yet this abatement of violence will not prevent their actual continuance from progressively diminishing the energies of life, and eventually terminating the existence of the patient. Mr. H. would do well to recollect, amidst the subtilties of hypothesis, that though life is supported by stimulating agency, yet that the excesses and irregularities of excitement are the common causes of death, and that whatever may have occasioned a departure from the standard of health, whether such departure should consist in an excessive or deficient action of vital power, a prompt restoration of that standard is the eligible mode of cure. The removal of diseased action is in no instance likely to be too speedy. The state of disease indeed is not susceptible of so instantaneous a conversion to health as could involve much risk even on the Brunonian, or the expositor, Mr. H's scheme of graduated stimulation.

Mr. H. expresses his surprise at my confounding what he calls "the *transient* application of a powerful exciting cause, and one that *continues* to operate with undiminished force," as is the case in instances of inflammatory disease. But is not this making a distinction where there is no difference? What cause of inflammation can operate with more violence than the external application of spirit of turpentine conjoined with the internal exhibition of stimulating medicines? Such treatment, in my expectation, would not be so "*transient*" as Mr. H. imagines, but would, in my estimate of its probable action, generate a state of excitement that would "continue to operate with undiminished force," as in the instance of original inflammation,

Mr. H. objects to my asserting, that diseases of direct debility require "liberal excitement," saying, that "this doctrine is any thing rather than Brunonian." It is not my purpose strictly to define and vindicate either what is or what is not Brunonian, but to deliver my own sentiments respecting the actions of organic life. The idea of accumulated excitability resulting from the extreme weakness or comparative inaction attending an insufficient application of stimulating agents, has ever been in my judgment much too visionary to admit of being substantiated. If through deficient nutriment, excessive cold, the debilitating influence of immoderate grief, or the sedative operation of any other cause, the powers of life should be reduced to a low ebb, will it be said that vital energy or excitability has gone on to amass in such circumstances, that it has not been expended, and therefore must be redundant? This would be to expect the production of an effect without a cause. A *negative* condition of vital power cannot be *positively* active. The generating and amassing of vital power is the function of health, not of disease. All cases of debility, whether directly or indirectly induced, are marked by a deficiency of active energy; a tremulous irregularity in the movement of every fibre has superceded on such occasions, the equable and unyielding tone of healthful power. The natural renewal of strength after fatigue, on discontinuing the exertion which had tired, results from a salutary process founded on the innate resources of life, and does not obtain when disease interferes with its regular performance. The mode of recruiting strength in cases of direct, as well as indirect debility, is to furnish the genial excitement afforded by nutritious support, and to remove, as soon as possible, the weakening influence of diseased action. By this mode strength may be restored, not indeed by exhausting superfluous excitability, but by actually generating the healthful power of life. That the torpor of disease should spontaneously terminate in an exuberance of vital power is much less probable, than that it should proceed to palsy and death, if not seasonably obviated by nutritive and other salubrious stimulants.

It is not my wish to treat any opinion of Mr. H. with levity or neglect; his candour is entitled to my high respect, and warrants me in presuming that he will indulge me in enjoying my own persuasion in physiological subjects, until it shall be controverted by an irresistible shew of reason, and of facts. I am, &c.

*Taunton, May 22, 1806.* ROBERT KINGLAKE.

